# Epigenetic Therapy of Non-Small Cell Lung Cancer Using Decitabine (5-Aza-2′-Deoxycytidine)

**DOI:** 10.3389/fonc.2013.00188

**Published:** 2013-07-29

**Authors:** Richard L. Momparler

**Affiliations:** ^1^Département de Pharmacologie, Centre de Recherche du CHU Sainte-Justine, Université de Montréal, Montreal, QC, Canada

**Keywords:** non-small cell lung cancer, chemotherapy, decitabine, epigenetics

## Abstract

Epigenetic analysis shows that many genes that suppress malignancy are silenced by aberrant DNA methylation in lung cancer. Many of these genes are interesting targets for reactivation by the inhibitor of DNA methylation, decitabine (5-aza-2′-deoxycytidine, DAC). A pilot study on intense dose DAC showed promising results in patients with metastatic non-small cell lung cancer (NSCLC). However, subsequent clinical studies using low dose DAC were not very effective against NSCLC and interest in this therapy diminished. Recently, interesting responses were observed in a patient with NSCLC following treatment with a combination of the related inhibitor of DNA methylation, 5-azacytidine, and an inhibitor of histone deacetylation. This finding has generated a renewed interest in the epigenetic therapy of lung cancer. Preclinical studies indicate that DAC has remarkable chemotherapeutic potential for tumor therapy. This epigenetic agent has a delayed and prolonged epigenetic action on tumor cells. This delayed action should be taken into consideration in the design and evaluation of clinical studies on DAC. Future research should be directed at finding the optimal dose-schedule of de DAC for the treatment of NSCLC.

## Introduction

The life expectancy of most patients with metastatic non-small cell lung cancer (NSCLC) following standard chemotherapy is very limited (1–3). There is an urgent need to develop more effective treatments for this malignancy. Molecular changes that place in the genome of lung cancer cells can give insight to novel targets for chemotherapeutic intervention. Analysis of the genome of lung cancer shows that both genetic and epigenetic events are implicated in tumorigenesis. It is not possible to reverse the genetic aberrations with current chemotherapy. However, epigenetic changes are reversible and interesting targets for chemotherapeutic intervention. One of the major epigenetic changes that take place in lung cancer is aberrant DNA methylation in the promoter region of genes that suppress malignancy. The silencing of these genes can promote tumorigenesis by several mechanisms including increasing cell proliferation, inactivation of genes that suppress metastasis and angiogenesis, suppressing apoptosis, and DNA repair. These genes are interesting targets for intervention using a potent inhibitor of DNA methylation, such a decitabine (5-aza-2′-deoxycytidine, DAC).

## Pharmacology of Decitabine

5-Aza-2′-deoxycytidine is an analog of deoxycytidine and a prodrug that has to be activated by photophosphorylation by deoxycytidine kinase (4). Since its antineoplastic action is dependent on its incorporation into DNA, DAC is an S phase specific agent. The incorporation of DAC in place of 5-methylcytosine in DNA results in the inactivation of DNA methyltransferase 1 due to covalent bond formation between the 5-azacytosine ring of DAC and this enzyme. The end result of this process is hypomethylation of DNA. Genes that are silenced by aberrant DNA methylation can be reactivated by treatment with DAC. Both leukemic and tumor cells are very sensitive to the antineoplastic action of low concentrations of DAC. In animal models of leukemia and cancer, DAC shows curative potential (5). The antineoplastic action of DAC is very dose-schedule dependent. The major side effect of DAC is granulocytopenia.

## Pilot Study on DAC in Patients with Advanced NSCLC

A phase I–II clinical trial on DAC was performed at our University medical center on patients with stage IV NSCLC who had received no prior chemotherapy (6). The patients received an 8-h i.v. Infusion of DAC at intense doses of 200–660 mg/m^2^. For five patients that received 2–4 cycles of DAC, three patients survived 4.3–9.3 months, and two patients survived 15.3 and 16 months, respectively. (7). One patient that received five cycles of DAC survived 81 months. This latter patient was removed from the DAC study due to tumor progression and was treated with three cycles of vindesine over 15 month interval. She then received no further chemotherapy and eventually expired due to tumor progression. Due to the lack of understanding of the delayed action of DAC some patients were removed from the clinical study due to signs of tumor progression. The estimated steady state plasma concentration of DAC during the infusion of 660 mg/m^2^ in this patient was 2.6 μM. Due to the small sample size many oncologists may consider these interesting responses with intense dose DAC in patients with NSCLC as only anecdotal. This conclusion is supported by clinical trial using low dose DAC in patients with advanced NSCLC (8). Sixteen patients with stage IV NSCLC, adenocarcinoma diagnosis, were administered DAC as 72-h infusion at doses of 60–90 mg/m^2^ total. Most patients received two cycles of DAC. The overall response for all the patients was progressive disease. The estimated plasma concentration of DAC for these patients was in the range of 0.04–0.06 μM, which is 40- to 60-fold lower than observed with intense dose DAC.

## Analysis of Study on Intense Dose DAC in Patients with NSCLC

The key question with respect to intense dose DAC for the treatment of NSCLC is the observed responses an indication of its chemotherapeutic potential or just chance occurrence that rarely takes place. It is essential to look at both the clinical and preclinical data on an experimental drug before making a decision to continue or abandon future clinical investigations. An example of how this process can take place is described below on different key parameters that should be taken into account. One key mistake that should be avoided is to conclude that an experimental drug does not merit additional investigation based on the data from a clinical trial that used a very poor and suboptimal dose-schedule for the drug.

### Molecular mechanism of action

5-Aza-2′-deoxycytidine has a novel mechanism of action of reactivating genes that prevent or suppress malignancy. A list of some of the target genes is shown in Table 1. It is possible that there are many other genes that suppress malignancy that are also reactivated by DAC, but still have not been identified. Since DAC has many gene targets in malignant cells, does it have more therapeutic potential than an agent that targets only a single gene? Future investigations will answer this question.

**Table 1:** **Tumor suppressor genes silenced by aberrant DNA methylation in lung cancer**.

Gene	Function	Reference
*BHLHB4*	Transcriptional regulator, differentiation	(23)
*BLU*	Transcription repressor	(17, 23)
*CDKN2A*	Inhibitor cyclin-dependent kinase; cell cycle arrest	(15, 16)
*CDH1*	Cell adhesion/metastasis	(16)
*CDH13*	Cell adhesion	(16)
*CXCL14*	Pro-apoptosis, cell cycle arrest	(24)
*DAPK1*	Apoptosis	(16)
*DKK3*	Wnt pathway antagonist	(21)
*DCL1*	Cell growth, apoptosis, cell adhesion	(15)
*HOXA genes*	Homeobox transcriptions factors; differentiation	(22)
*MGMT*	DNA repair	(16, 23)
*MMP2*	Degradation extracellular matrix	(23)
*RARß*	Retinoic acid receptor ß; differentiation	(16)
*RASSF1A*	Cell cycle control	(16, 17, 25)
*Reprimo*	Mediator of p53-mediated cell cycle arrest	(20)
*RUNX3*	Transcription factor target TGF-ßpathway	(25)
*SFRP1*	Antagonist of Wnt pathway	(26)
*TCF1*	Differentiation	(19)
*TIMP3*	Tissue inhibitor of metalloproteinase 3; metastasis	(15)
*WIF1*	Antagonist of WNT pathway	(18)

### Therapeutic concentration and duration of exposure

The *in vitro* studies on the antineoplastic activity of DAC on human lung carcinoma cells provide the data on what concentration and exposure time is required to reduce colony formation. DAC at 0.44 μM for 8 h exposure reduced colony formation of SK-MES-1 and NCI-H520 lung carcinoma cells by 47.3 and 21.7%, respectively (6). A 10-fold higher concentration of 4.4 μM of DAC reduced colony formation by 76% for both cell lines. In the mouse tumor model the infusion of DAC for 18-h to give a steady state plasma level of 2.9 μM produced a 2-log cell kill of the tumor cells (5). In comparison, the patient who survived>5 years received an 8-h infusion of DAC at a dose of 660 mg/m^2^, which produced a plasma level was 2.7 μM. This interesting correlation shows that in the clinical trial with intense dose DAC, concentrations of this analog were present in the plasma of the patients that had the potential to significantly reduce the clonogenic potential of the tumor cells.

### Duration of treatment

The *in vitro* colony assay shows that the longer exposure time of 24-h was more effective than the 8-h exposure. DAC at 4.4 μM exposure for 24-h produced a>90% reduction in colony formation for both the lung carcinoma cell lines (6). In a mouse tumor model a 24-h infusion of DAC was much more effective than a 6- or 12-h infusion (9). This shows that the 8-h infusion used in the clinical study was clearly suboptimal, but was chosen to avoid the risk of unacceptable toxicity for the first patients that entered the phase I trial. The preclinical data support the use of longer infusion times of DAC in future clinical trials in patients with NSCLC.

## Design of Clinical Trials on DAC in Patients with NSCLC

It is not justified to conclude that DAC is weak antitumor drug based on the results of clinical trial that used very poor dose-schedule? In order to determine the full chemotherapeutic potential of DAC for the treatment of lung cancer, it is important to find its optimal dose-schedule. Several suggestions are summarized here for the design of a clinical trial on DAC in patients with NSCLC with the long-term objective of finding its optimal dose-schedule for cancer treatment.

### Patient selection

The major toxicity produced by DAC is myelosuppression. The patients with NSCL that are candidates for a clinical trial on intense doses of decitabine should have a good performance and hematologic status. A minimum of 4 weeks after previous cytotoxic chemotherapy is necessary to permit adequate recovery from bone marrow toxicity. An interesting cohort of NSCLC patients would be those who were previously treated with targeted agents (Erlotinib, Gefitinib), which produce no hematotoxicity.

### Dose of DAC

One of the major reasons for failure of cancer chemotherapy is the limited penetration of cytotoxic drugs into tumors (10). Due to the low therapeutic index of most cytotoxic anticancer drugs, there is a limit that one can increase the dose to obtain therapeutic concentrations in the central region of the tumor microenvironment. In this regard, the S phase specificity of DAC permits the use of high doses of this agent for cancer treatment. The scientific rationale for the use of intensive doses of DAC is based on its comparative pharmacology to ARA-C. Both these agents are analogs of deoxycytidine, have an identical metabolism and their antineoplastic action is due to their incorporation in DNA. However, their mechanisms of action are different. ARA-C is a potent inhibitor of DNA replication, whereas DAC is potent inhibitor of DNA methylation. In clinical trials in patients with leukemia the standard ARA-C dose of 100 mg/m^2^/day was increased to 6,000 mg/m^2^/day (11). This represents a 60-fold increase in the total dose per day for ARA-C. Since both deoxycytidine analogs target the same S phase cells, their profiles for hematopoietic toxicity are similar. The preclinical data predict that concentrations of DAC>1 μM are required to obtain a potent antitumor effect. This drug should be infused at a rate that gives this level in the plasma. For example, an infusion rate of 50 mg/m^2^/h of DAC gives a steady state plasma concentration for DAC in the range of 2 μM.

### Duration of DAC infusion

The preclinical data clearly show that a 24-h drug exposure is much more effective than an 8-h exposure of DAC against human lung carcinoma cells (6). Without question the conservative 8 h infusion that was used in our pilot clinical study on lung cancer was too short to produce good responses in all patients. A good starting dose-schedule to use for DAC in a clinical trial in patients with metastatic NSCLC would be 50 mg/m^2^/h for 18 h for a total dose of 900 mg/m^2^. The key objective of this dense dose DAC therapy is to eradicate the proliferative potential of the most rapidly growing tumor stem cells. Depending on the response and adverse events, the rate of drug infusion rate can be kept constant and the duration of the infusion increased by intervals of 6 h.

### Duration between cycles of DAC therapy

Myelosuppression is the major toxicity produced by DAC. Due to the delayed epigenetic action of DAC on hematopoietic cells, the recovery from granulocytopenia takes about 2 weeks longer than that observed with cytotoxic chemotherapy, such as ARA-C (12). Many centers used a 4-week interval between cycles of cytotoxic chemotherapy. In the case of DAC, it is recommended to use a 6-week interval between cycles so as to permit a more full recovery of the granulocyte count. The delayed epigenetic action of DAC also takes place on the tumor cells, which results in a slower recovery of their growth potential as compared to cytotoxic agents, which also permits a longer interval between cycles of chemotherapy.

### Monitor of clinical response

Tumor cells treated with DAC undergo several cell divisions before growth arrest due to the time this epigenetic agent takes to reactivate the expression of silent tumor suppressor genes. Without a sound understanding of the delayed epigenetic action of DAC, one can conclude that the tumor is resistant to this agent. An increase in tumor size of ≥20% after drug treatment is an indication for removal of the patient from the clinical study at many treatment centers. It is suggested that this criteria for patient removal from the study should be increased to ≥50% for DAC. This suggestion should be confirmed by careful evaluation and may have to be modified depending on long-term clinical response.

### Number of cycles of DAC treatment

The patient with NSCLC that survived 81 months in our pilot study received five cycles of DAC (6). It is recommended that patients with good performance status also be administered five cycles of intense dose DAC.

## Conclusion

The epigenetic agent DAC shows considerable promise for the treatment of NSCLC. The interesting response observed in a patient with NSCLC following treatment with a combination of inhibitors of DNA methylation and histone deacetylation is an indication of that positive results can be obtained with epigenetic agents (13). This latter study confirms both our preclinical and clinical studies on DAC (6, 14). A good understanding of the pharmacology of DAC and its delayed epigenetic action on tumor cells should be taken into consideration in the design of clinical trials on patients with advanced NSCLC (4, 9, 12). The full chemotherapeutic potential of epigenetic therapy of cancers will require intensive clinical investigation (5). Table 1 summarizes the large number tumor suppressor genes that are silenced by aberrant DNA methylation in NSCLC (15–26) and which are interesting targets for DAC therapy. These observations provide a positive outlook for future progress for the therapy of lung cancer.

## Conflict of Interest Statement

The author declares that the research was conducted in the absence of any commercial or financial relationships that could be construed as a potential conflict of interest.
